# The Palaeozoic assembly of the holocephalan body plan far preceded post-Cretaceous radiations into the ocean depths

**DOI:** 10.1098/rspb.2024.1824

**Published:** 2024-10-30

**Authors:** Chase D. Brownstein, Thomas J. Near, Richard P. Dearden

**Affiliations:** ^1^ Department of Ecology and Evolutionary Biology, Yale University, New Haven, CT 06511, USA; ^2^ Yale Peabody Museum, New Haven, CT 06511, USA; ^3^ Vertebrate Evolution, Development, and Ecology, Naturalis Biodiversity Center, Darwinweg 2, Leiden 2333 CR, The Netherlands; ^4^ School of Geography, Earth & Environmental Sciences, University of Birmingham, Edgbaston, Birmingham B15 2TT, UK

**Keywords:** *Holocephali*, phylogenetics, chimaeras, extinction, fossil, deep sea

## Abstract

Among cartilaginous fishes, *Holocephali* represents the species-depauperate, morphologically conservative sister to sharks, rays and skates and the last survivor of a once far greater Palaeozoic and Mesozoic diversity. Currently, holocephalan diversity is concentrated in deep-sea species, suggesting that this lineage might contain relictual diversity that now persists in the ocean depths. However, the relationships of living holocephalans to their extinct relatives and the timescale of their diversification remain unclear. Here, we reconstruct the evolutionary history of holocephalans using comprehensive morphological and DNA sequence datasets. Our results suggest that crown holocephalans entered and diversified in deep (below 1000 m) ocean waters after the Cretaceous–Palaeogene mass extinction, contrasting with the hypothesis that this ecosystem has acted as a refugium of ancient cartilaginous fishes. These invasions were decoupled from the evolution of key features of the holocephalan body plan, including crushing dentition, a single frontal clasper, and holostylic jaw suspension, during the Palaeozoic Era. However, these invasions considerably postdated the appearance of extant holocephalan families 150 million years ago during a major period of biotic turnover in oceans termed the Mesozoic Marine Revolution. These results clarify the origins of living holocephalans as the recent diversification of a single surviving clade among numerous Palaeozoic lineages.

## 1. Introduction

The *Holocephali* is by far the most species-poor and morphologically conservative of the four constituent clades of jawed vertebrates (gnathostomes), the others being elasmobranchs, sarcopterygians and actinopterygians [1]. Owing to their relatively small genome sizes compared to other cartilaginous vertebrates [2], holocephalans, which include the chimaeras, elephant sharks and their relatives, have provided a wealth of information on early gnathostome evolution. Several extinct clades of vertebrates from the Palaeozoic with classically recalcitrant relationships [3,4] seem to show affinities to holocephalans [1,5–11]. *Holocephali* has provided important clues about the early evolution of jawed vertebrates [1,5,12–15]. A combination of expanding genomic resources [2] and anatomical data from exceptionally preserved fossil members of this clade [1,5–7,15,16] has illuminated the complex early evolution of the characteristic skeletal, soft tissue and physiological characteristics during the initial radiation of the jawed vertebrate crown group.

Holocephalan diversity is currently concentrated in oceanic environments below depths of 200 m [17–21]. Given their ancient common ancestry with other vertebrates, deep water chimaeras and ghost sharks lend credence to the hypothesis that mesopelagic and bathypelagic environments have served as a refuge for ancient biodiversity that has become rare or extinct in other ecosystems [22,23]. Evidence from the fossil record [24] and phylogenomic analyses of diverse living deep-sea vertebrate clades [25–33] suggest younger origins for these deep water radiations. Thus, chimaeras and ghost sharks stand as one of the remaining potential relict vertebrate lineages that have survived in the mesopelagic and bathypelagic zones.

Despite the importance of holocephalans for outstanding questions in evolutionary biology, the age of their living species diversity has only been inferred a handful of times using taxonomically limited samples [34–36], and the relationships of bizarre Mesozoic holocephalans [37–39] among the living clades and the numerous lineages known from the Palaeozoic remain unexplored in the twenty-first century [3]. These factors have obscured the origins of the specialized anatomy of living species [15,17] among morphologically disparate extinct holocephalans and the timescale of evolution in these early-diverging jawed vertebrates.

Here, we reconstruct the evolutionary relationships and timescale of divergence of crown holocephalans and their extinct relatives using newly assembled data from the fossil record and DNA sequences from 81% (35/47) of the extant species diversity of the clade [40]. Our results establish and contextualize a Jurassic origin of crown *Holocephali* among numerous Palaeozoic stem holocephalan clades, followed by the survival of only two lineages through the end-Triassic mass extinction. Although we find support for a rapid assembly of key features defining the crown holocephalan body plan during the Palaeozoic evolution of the total clade, we infer with perhaps one exception that all crown deep-sea chimaera and ghost shark clades appeared and diversified following the Cretaceous–Palaeogene mass extinction 66.02 million years ago [41]. This recent origin for most deep water holocephalans eliminates the last major contender for a deep-sea ‘living fossil’ vertebrate diversity and establishes this environment as a source of both species richness and morphological innovation [20,42] in this lineage.

## 2. Methods

### (a) Systematics

In this study, we follow the conventions of the PhyloCode, which was recently formally applied to ray-finned fish systematics [43], for describing clades [44]. In practice, this means that we refer to total clades using the prefix ‘pan-’. We also follow emerging conventions in italicizing all clade names [43–45].

### (b) Morphological dataset construction

In order to test the phylogenetic position of living holocephalans and Mesozoic species among the larger Palaeozoic diversity of the total clade, we constructed a new taxon–character matrix by expanding the dataset of Frey *et al*. [8] using characters from Didier [17] and Patterson [46]. We also edited character states and added 14 living and extinct holocephalan taxa to thoroughly sample holocephalan diversity within and proximal to the crown clade. Newly added data include genus-level scorings for all five living holocephalan genera, as well as for species in the genera †*Acanthorhina*, †*Chimaeropsis*, †*Elasmodectes*, †*Ischyodus*, †*Metopacanthus*, †*Myriacanthus* and †*Squaloraja*. Owing to the lack of available information on their morphology and our focus on incorporating post-Palaeozoic taxa into the analysis, we did not include any representatives of the Palaeozoic pan-holocephalan clades †*Eugeneodontida* and †*Petalodontiformes*, which mostly include tooth taxa and for which only a handful of holomorphic specimens [10,11,47] of limited phylogenetic informativeness are known. Future discoveries, including the description of unpublished specimens [4], will be needed to resolve the relationships of these Carboniferous and Permian pan-holocephalans; we expect that the phylogenetic matrix employed in this study will contribute to this pursuit. The final matrix included 36 operational taxonomic units coded for 236 characters. Details of sources for character scorings, character additions, removals, state modifications and taxon inclusion are included in the electronic supplementary material.

### (c) DNA sequence dataset assembly

In order to sample the species diversity of living holocephalans most fully, we targeted sequences for the *cytb*, *COXI*, *ND2* and *16*s mitochondrial loci on the NCBI repository GenBank. Using this approach, we were able to sample 100% of all living holocephalan genera and species complexes, including all three species in *Callorhinchus*, seven of the nine recognized species in *Rhinochimaeridae* and 35 of 47 described species of *Chimaeridae* [40], plus two specimens of *Chimaera* sp. with mitogenomes that appear to diverge considerably from other species [48,49]. We concatenated these data into a single set of aligned sequences for subsequent phylogenetic analyses.

### (d) Morphological phylogenetic analyses

We analysed the morphological matrix using a maximum parsimony approach in PAUP* v. 4.0a [50]. We rooted the tree on the maxillate ‘placoderm’ stem-gnathostome †*Entelognathus primordialis* [51,52] and ran a heuristic search with tree bisection–reconnection (TBR) branch swapping and 1000 addition sequence replicates holding five trees from each replicate (electronic supplementary material, figure S1). We also analysed the morphological matrix using a Bayesian approach in MrBayes v. 3.2.7a [53], with trees rooted on †*Entelognathus*. We used an Mk_v_ model with a gamma-distributed rates parameter, ran the analysis for 3.0 × 10^6^ generations, sampled every 1000 generations and combined the trees with a burnin of 25% following confirmation of chain convergence using a standard deviation of split frequencies of less than 0.01 and confirming adequate mixing in Tracer v. 1.7.1 [54]. Trees resulting from the Bayesian analysis were summarized using a 50% majority rule consensus topology.

Next, we conducted a Bayesian tip-dating analysis of our morphological dataset and a revised set of tip dates for included extinct species (see electronic supplementary material) under the fossilized birth–death (FBD) model [55] as implemented in BEAST v. 2.6.7 [56,57] with the Mk_v_ model of morphological evolution [58]. We again rooted trees on †*E. primordialis* and set the origin prior to 443.8 Ma (Ordovician–Silurian boundary), with bounds of 514.1 Ma (median age of crown vertebrates found by two recent genomics papers [59,60]) and 439.0 Ma (the age of the oldest definite crown gnathostome [61]), as no crown vertebrates are known before Cambrian Stage 3 [62] and crown gnathostomes appear to have initially diversified in the middle–late Ordovician based on both genomic [59,60] and morphological [61,63–68] phylogenies. We conducted two independent runs over 1.0 × 10^8^ generations with 1.0 × 10^7^ pre-burnin and checked for convergence of the posteriors and effective sample size values over 200 using Tracer v. 1.7.1 [54]. The resulting posterior tree sets were combined in LogCombiner 2.6.7 with 10% burnin [56] each into a maximum clade credibility tree with median node heights in TreeAnnotator v. 2.6.6 [56].

### (e) Molecular phylogenetic analyses

We conducted maximum likelihood inference on the concatenated sequence alignment using IQ-TREE [69] on the online web server, given the size of the dataset [70]. We calculated standard bootstraps over 100 replicates and Shimodaira–Hasegawa-like (SH) approximate likelihood ratio tests over 1000 replicates to assess support for given nodes. Next, we conducted Bayesian tip-dating phylogenetic analysis of the sequence alignment in BEAST v. 2.6.6 using the FBD model and including stem-holocephalan and extinct crown holocephalan clades as fossil tip calibrations, with their placements constrained using monophyletic most recent common ancestor (MRCA) priors following the results of Bayesian tip-dating analysis of the morphological dataset. We ran the analysis three times independently over 1.0 × 10^8^ generations, each with a 5.0 × 10^7^ pre-burnin, combined posterior tree sets in LogCombiner v. 2.6.7 after checking for convergence of the posteriors and effective sample size (ESS) values over 200 in Tracer v. 1.7.1 following 25% burnin and summarized the trees in a single maximum clade credibility tree with median node heights in TreeAnnotator v. 2.6.6.

### (f) Ancestral state reconstructions

Using our tip-dated phylogeny of living holocephalans, we conducted ancestral state reconstructions of adult habitat preference and several features of the living holocephalan body plan to understand the timescale of assembly of the morphology of the crown clade and the origin of deep-sea lineages in time. We ran all ancestral state reconstructions using the R package phytools [71]. We collected anatomical data from the literature [1,3,8,14,15,17], examination of CT scan data (electronic supplementary material) and data on habitat occupation from the FishBase database (https://www.fishbase.se/). Habitat levels are based on previous studies examining depth occupation through deep time by animals [31,72]: 0–200 m was coded as epipelagic, 200–1000 m was coded as mesopelagic and below 1000 m was coded as bathypelagic. To accommodate the observation that many living holocephalans occupy multiple regions of the water column, we used a symmetrical polymorphic character model using the polyMk function in phytools. In our ancestral state reconstructions of habitat and key anatomical features, we conducted simulated stochastic mapping over 1000 simulations using the tip-dated phylogeny focusing on living holocephalans and summarized the posterior distribution of reconstructed ancestral states along a single tree.

## 3. Results

### (a) Phylogenetic analyses of the morphological matrix

**Figure 1.**
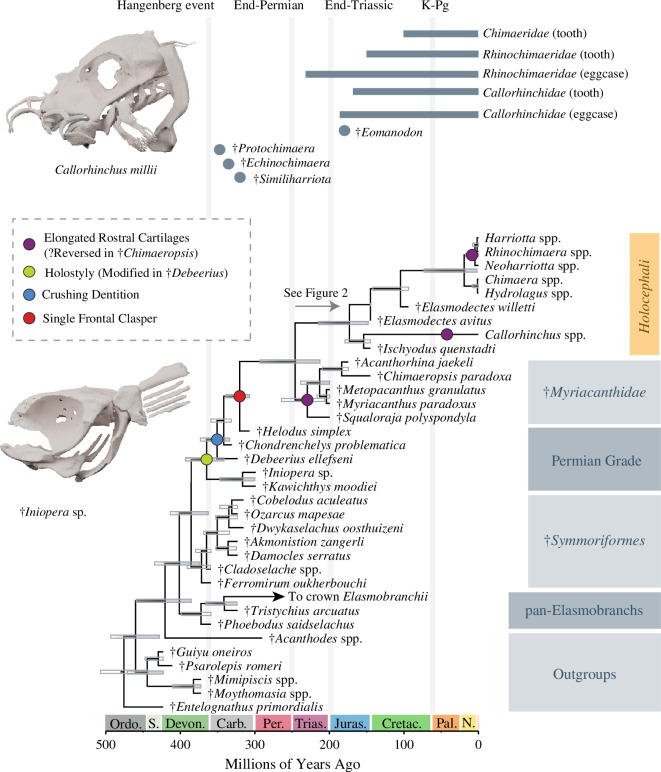
Tip-dated Bayesian phylogeny of living and extinct holocephalans based on morphological characters. Maximum clade credibility tree generated from Bayesian analysis of the morphological matrix and fossil ages in BEAST v. 2.6.7. Bars at nodes represent 95% HPD intervals for divergence times, and clear bars denote nodes with posterior support values of less than 0.80. Coloured dots at nodes indicate inferred origins for key holocephalan features, and computed tomography scan renders spotlight living and extinct holocephalan morphology. Dots above the phylogeny represent the ages of putative crown holocephalans and records of toothplates and eggcases associated with major lineages in the crown group (electronic supplementary material). K-Pg = Cretaceous-Palaeogene Mass Extinction.

Our analyses of the 236 character matrix (figure 1; electronic supplementary material, figures S2) resolved four major clades among total-group *Holocephali*, which our Bayesian tip-dated phylogeny estimates diverged from other chondrichthyans 401.41 million years ago (95% highest posterior density (HPD): 374.52, 434.44 Ma) in the early Devonian. Our phylogeny of *Holocephali* largely agrees with previous analyses [8] regarding the relationships of Palaeozoic lineages. The relationships we recover for holocephalans are also broadly consistent across the three analytical approaches (parsimony, uncalibrated Bayesian and tip-dated Bayesian analysis). The first holocephalan clade to diverge, †*Symmoriformes*, includes Devonian and Carboniferous taxa [1,8]. Bayesian analyses incorporating (figure 1) or excluding (electronic supplementary material, figure S2) fossil age data resolve the monophyly of this clade with weak to moderate posterior support and place the MRCA of species included in our phylogeny at 371.59 Ma (95% HPD: 359.84, 390.76 Ma). Next to diverge across all analyses are the †*Iniopterygiformes*, an assemblage of marine chondrichthyans with highly unusual body plans that have historically been difficult to place among jawed vertebrates [4,7,9,15]; in our phylogeny, this clade last shares an MRCA with other holocephalans 364.69 Ma (95% HPD: 341.4, 392.77 Ma), in the latest Devonian (figure 1). As in two recent studies [5,8], we infer that †*Kawichthys moodiei* is more closely related to †*Iniopera* than to †*Symoriiformes* [6].

We resolve the species †*Chondrenchelys problematica* [73] and †*Helodus simplex*—the former of which may represent a larger clade of middle–late Palaeozoic species with ethmoid claspers [4,73]—in the same positions as found in previous analyses of the Frey *et al*. [8] dataset. †*Chondrenchelys* is placed closer to the crown clade than †*Debeerius ellefseni*, which appears far more similar to living chimaeras in general body shape [74]. Ancestral state reconstructions of key features of living holocephalans, including the single frontal clasper and durophagy, show that these features first appeared within this grade in the Palaeozoic over a period of approximately 40 million years (figure 1). †*Helodus simplex* is sister to a long branch extending through the Permian that leads to the two major Mesozoic–Cenozoic holocephalan clades crown *Holocephali* and †*Myriacanthidae*, which we estimate diverged in the Early Triassic 245.97 Ma (95% HPD: 212.66, 239.19 Ma), approximately 5 million years after the end-Permian mass extinction. The monophyly of the clade containing crown *Holocephali* and †*Myriacanthidae* is resolved across all analyses with strong bootstrap and posterior support (figure 1; electronic supplementary material, figures S2). The crown clade contains species in the living lineages *Callorhinchidae*, *Chimaeridae* and *Rhinochimaeridae* and the extinct genera †*Ischyodus* and †*Elasmodectes* and is supported with strong bootstrap and posterior support values. The MRCA of the crown clade in our tip-dated Bayesian phylogeny generated using morphological data is placed at 173.14 Ma (95% HPD: 147.94, 214.47 Ma). We resolve the Jurassic species †*Squaloraja polyspondyla*, †*Myriacanthus paradoxus*, †*Metopacanthus granulatus*, †*Acanthorhina jaekeli* and †*Chimaeropsis paradoxa* as a clade across all analyses with weak support. The oldest available name for this clade is the †*Myriacanthidae* [46].

### (b) Phylogenetic analyses of the DNA sequence dataset

**Figure 2.**
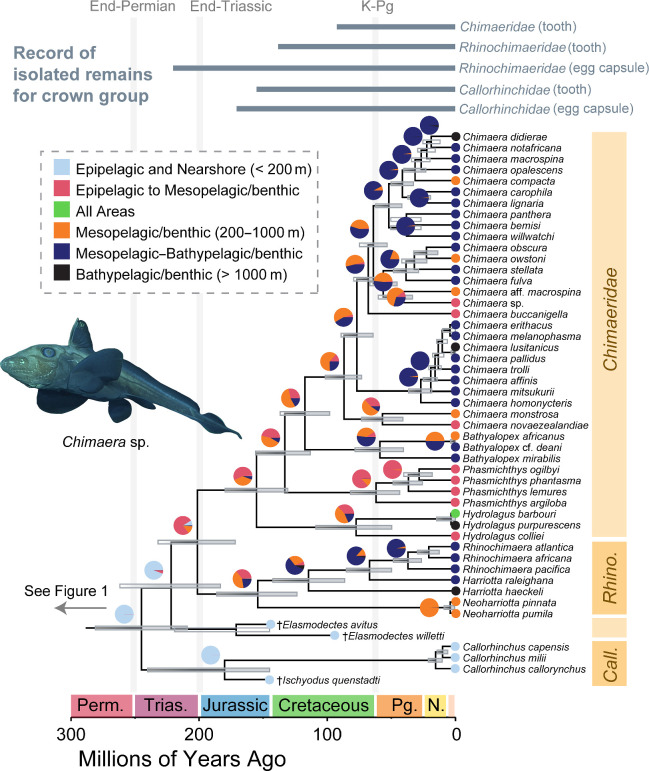
Tip-dated Bayesian phylogeny of living holocephalans. Maximum clade credibility tree generated from Bayesian analysis of the DNA sequence dataset and fixed fossil tip calibrations in BEAST v. 2.6.7. Bars at nodes represent 95% HPD intervals for divergence times, and clear bars denote nodes with posterior support values of less than 0.80. Pie charts at nodes show reconstructed probabilities for given habitat states, and circles at tips show states associated with each species. Bars above the phylogeny spotlight records of toothplates and eggcases associated with major lineages in the crown group (see electronic supplementary material). The photograph of *Chimaera* sp. is in the public domain from the National Ocean and Atmospheric Administration (NOAA). Abbreviations: *Call*., *Callorhinchus*; *Rhino.*, *Rhinochimaeridae*.

Our analyses of the molecular sequence alignment delimit three major clades of living holocephalans following previous studies [17,34,35]: the elephant sharks in *Callorhinchus*, the longnose chimaeras and spookfishes in *Rhinochimaeridae* and the chimaeras, rabbit fishes, rat fishes and ghost sharks in *Chimaeridae* (figure 2; electronic supplementary material, figure S3). Although *Chimaeridae* and *Rhinochimaeridae* are found to be sister clades in all analyses (cf. [34,35]), this relationship is weakly supported in both maximum likelihood and Bayesian phylogenies (figure 2; electronic supplementary material, figure S3). *Rhinochimaeridae* includes the genera *Harriotta*, *Neoharriotta* and *Rhinochimaera*, the former of which is found to be paraphyletic with respect to *Rhinochimaera* (figure 2; electronic supplementary material, figure S3). *Chimaeridae* includes species traditionally placed in the genera *Chimaera* and *Hydrolagus* (figure 2; electronic supplementary material, figure S3). However, as in previous studies, we fail to resolve the reciprocal monophyly of these genera (electronic supplementary material, figure S3) [34,49,75,76]. Instead, we resolve four major clades in *Chimaeridae*, two of which necessitate the resurrection of available generic names.

The first clade to diverge from other chimaerids is *Hydrolagus*, which includes the type species *H. colliei* and the species *H. barbouri* and *H. purpurescens* (figure 2; electronic supplementary material, figure S3). Next to diverge is a clade containing ‘*Chimaera*’ *argiloba*, ‘*C*.’ *phantasma*, ‘*C.*’ *ogilbyi* and ‘*H*.’ *lemures* (figure 2; electronic supplementary material, figure S3). ‘*Hydrolagus*’ *lemures* is the type species of the genus *Phasmichthys* [77], which is the oldest available name for this lineage; we resurrect this genus to include these four species (figure 2; electronic supplementary material, figure S3). We also resurrect the genus name *Bathyalopex* [78] for a clade containing ‘*H. mirabilis*’ (the type species of *Bathyalopex*), ‘*H*.’ *africanus* and ‘*H*.’ *deanii* (figure 2; electronic supplementary material, figure S3). This taxonomy restricts the genus *Chimaera* to a clade of at least 25 known species, including the type *C. monstrosa*. All these clades are reciprocally monophyletic, with strong support in our maximum likelihood and Bayesian phylogenetic analyses (figure 2; electronic supplementary material, figure S3).

We place the divergence of crown holocephalans from their closest relatives in the Early Triassic 245.06 Ma (95% HPD: 209.31, 280.94 Ma) in our tip-dated Bayesian phylogeny made using the DNA sequence dataset. Although it is the earliest diverging living lineage of holocephalans, we place the common ancestor of living species in *Callorhinchus* at 15.49 Ma (95% HPD: 10.31, 21.1 Ma). This result is congruent with the time tree obtained by Inoue *et al*. [35] and demonstrates that *Callorhinchus* represents the recent diversification of an exceptionally long branch extending from the earliest Mesozoic. In contrast, the clades *Chimaeridae* and *Rhinochimaeridae* last share common ancestry at the Triassic–Jurassic boundary 201.31 Ma (95% HPD: 171.72, 232.05 Ma) in our tip-dated phylogeny. We estimate that both *Chimaeridae* and crown *Rhinochimaeridae* appear in the Kimmeridgian Stage of the Late Jurassic at 155.2 Ma (95% HPD: 130.77, 179.73 Ma) and 154.18 Ma (95% HPD: 123.56, 186.16 Ma), respectively. Except for *Chimaera* and *Hydrolagus*, all chimaerid and rhinochimaerid genera resolved as monophyletic appear after the Cretaceous–Palaeogene mass extinction (figure 2). The major subclades in *Chimaera* also represent post-Cretaceous diversifications (figure 2).

### (c) Evolution of habitat preference

Our ancestral state reconstruction of habitat preference across holocephalan phylogeny broadly finds evidence for multiple recent deep-sea invasions and subsequent diversifications rather than an ancient history of life in the meso- and bathypelagic zones. The common ancestor of crown holocephalans is reconstructed as an epipelagic inhabitant with strong support, and it is only after the end-Triassic extinction that mesopelagic clades appear (figure 2). Except for a clade containing species in the genera *Harriotta* and *Rhinochimaera* with a mid-Cretaceous origin 114.5 Ma, the evolutionary history of bathypelagic habitation dates to after the Cretaceous–Palaeogene mass extinction in *Holocephali*. Indeed, our ancestral state reconstruction posits the early Cenozoic as an important period of ecological transition in chimaerid and rhinochimaerid fishes.

Following a transition to partially or fully mesopelagic lifestyles along the backbone of *Chimaeridae*, four major clades transition to the bathypelagic zone. One is *Bathyalopex* (MRCA age: 62.02 Ma). The other three ancestrally bathypelagic clades are species groups in *Chimaera*. The youngest includes the MRCA of *C. affinis* and *C. homonycteris* and all its descendants (MRCA age: 27.06 Ma), the second youngest includes all species sharing an MRCA with *C. fulva* and *C. obscura* (MRCA age: 38.53 Ma) and the oldest includes all species sharing an MRCA with *C. bemisi* and *C. opalescens* (MRCA age: 52.07 Ma). Notably, there is weak support for an ancestral bathypelagic habitat for the larger clade containing the latter two lineages (figure 2), which last shares a common ancestor 64.05 Ma (95% HPD: 53.29, 74.84 Ma) and is the sister lineage to *Chimaera buccanigella*. Among other major chimaerid lineages, the ancestral habitat of *Hydrolagus* species is unclear but likely in the mesopelagic and bathypelagic zones, and the ancestral habitat of *Phasmichthys* species is strongly inferred to be the mesopelagic and epipelagic zones (figure 2).

## 4. Discussion

Here, we have provided the first species-level phylogeny and hypothesis of diversification for the *Holocephali* (figures 1 and 2). Our results are consistent with the view that living holocephalan diversity represents one of two clades that emerged from a far more morphologically diverse grade of Palaeozoic lineages, including the †*Symmoriformes* and †*Iniopterygiformes* [1,46]. Together with the †*Myriacanthidae*, which includes extinct genera notable for their elongated rostral cartilages [79] and highly modified cephalic claspers [3,37], our phylogeny shows that crown *Holocephali* originated from a single clade that survived the Permo–Triassic mass extinction. Although there is some evidence from tooth fossils that representatives of †*Symmoriformes* persisted into the Cretaceous [80,81], the identity of these putative Cretaceous symmoriform teeth is controversial [82]. These results highlight the importance of the successive Permo–Triassic and end-Triassic mass extinctions for constraining the phylogenetic and morphological diversity of living holocephalans and their closest relatives.

Our age estimates for the holocephalan crown group are broadly consistent with the record of isolated teeth and egg capsules attributed to the *Chimaeridae*, *Rhinochimaeridae* and *Callorhinchidae* (figure 2; electronic supplementary material, table S1) [83,84]. The only major disagreement between the egg capsule fossil record and our inferred ages is the oldest described rhinochimaerid egg capsule (Carnian); this could imply that rhinochimaerids are older than we estimate or that rhinochimaerid egg capsule morphologies are either plesiomorphic for *Chimaeriformes* or were convergently evolved within the clade. Within the crown group, we resurrect two genera, *Bathyalopex* and *Phasmichthys*, for clades in *Chimaeridae* that diverged from recognized lineages in the Jurassic and Cretaceous (figure 2). This clarifies the systematics of living holocephalans, particularly confusion surrounding the classification of species traditionally placed in the chimaerid genera *Chimaera* and *Hydrolagus* [75,76]. Our placement of species in the genera †*Ischyodus* and †*Elasmodectes* on the stems of two lineages in crown *Holocephali* also provides a starting point for a reinterpretation of the articulated remains of putative crown group *Holocephali*, our understanding of which lags behind isolated tooth plates.

Previous studies of the phylogeny and timescale of evolution of holocephalans have focused solely on living species [34,35] or Palaeozoic fossils [1,5,8] to establish a timescale of chondrichthyan and holocephalan evolution. Our tip-dated Bayesian phylogeny of *Holocephali* infers that the divergence of living holocephalans from their closest relatives, the †*Myriacanthidae*, occurred just after the Permo–Triassic mass extinction. Several Carboniferous taxa have been attributed to the *Chimaeriformes*, i.e. in or closely related to the crown *Holocephali*, including the holomorphic †*Echinochimaera meltoni* [85] and †*Similiharriotta dabasinskasi* [86] and the toothplate taxon †*Protochimaera mirabilis* [87] (figure 1). Although we were unable to incorporate these taxa in our dataset, the antiquity of these species relative to our age estimates calls into question their placement within the holocephalan crown. The similar body shapes of crown holocephalans and Permo–Carboniferous stem-group taxa like †*D. ellefseni* (figure 1) suggest that similarities between †*Echinochimaera*, †*Similiharriotta* and living chimaeras may also have evolved convergently. However, based on our results, the Pleinsbachian tooth taxon †*Eomanodon simmsi* is credible as a close relative or early member of crown holocephalans [88].

Our results also place all family- and many genus-level divergences within the holocephalan crown clade and the †*Myriacanthidae* within a period of large-scale biotic change during the Early and Middle Jurassic called the Mesozoic marine revolution (MMR) [89–93]. Although the synchronicity of the Mesozoic diversification of holocephalans with the MMR follows the pattern of diversification in durophagous predator guilds during this event [90,91,93], our ancestral state reconstructions support a Palaeozoic origin for the crushing dentition of holocephalans as inferred in previous studies [1,73,74,94] and attested to by a rich fossil record of teeth from the Upper Devonian onward [95] within an earlier proliferation of durophagous clades [96].

The recent age of living holocephalan species diversity is also notable: 93% of living holocephalan species included in our tip-dated tree diverged from their closest relative in the past 65 million years. This pattern is especially evident in *Callorhinchus*, *Rhinochimaera*, *Neoharriotta* and the *Chimaera affinis–Chimaera pallidus* species group, which all originated between 2 and 27 million years ago (figure 2). Along with analyses of other clades with fossil records extending into the Palaeozoic, such as lampreys [97,98], our results highlight the recent origins of species diversity in some of the most deeply divergent vertebrate lineages.

The evolutionary history of *Holocephali* presented in this study adds to a growing body of evidence favouring a post-Jurassic origin of deep-sea vertebrate diversity. Our integration of fossil and molecular data (figures 1 and 2) supports a slightly older age for crown *Holocephali* than found in previous studies [35], but our ancestral state reconstructions show that holocephalans first invaded the bathypelagic zone in the Early Cretaceous. Indeed, we reconstruct most transitions into the deep sea occurring after the Cretaceous–Palaeogene mass extinction among species in the genus *Chimaera* (figure 2). These ages are comparable to the Cenozoic ages inferred for some species-rich deep-sea ray-finned fish clades, including anglerfishes [26,99], snailfishes and eelpouts [25,99,100], cods and grenadiers [99,101] and rockfishes [102], and even considerably postdate the ages of deep-sea transitions in the teleost clades *Aulopiformes* [28,30,31,103–105], *Stomiiformes* [33] and *Elopomorpha* [30,31,106–108]. Consequently, our study eliminates a key vertebrate lineage as a candidate relictual component of deep-sea assemblages. Instead, the time-calibrated phylogeny that we present here posits the Cretaceous–Palaeogene mass extinction as a facilitator of chimaerid diversification in the deep sea, perhaps in response to ecological opportunity [26,99,109,110]. Our results also establish that chimaeras probably invaded the bathypelagic zone multiple times, highlighting the complex habitat transitions that have occurred in this species-poor lineage over the past 66 million years of Earth’s history.

Cartilaginous fishes have been highlighted as a trove of ancient jawed vertebrate diversity [36]. Our results, which suggest that deep-sea holocephalan diversity originated relatively recently, show that this habitat, which now faces numerous anthropogenic threats [111–114], has been a cradle of species generation in a lineage containing over 400 million years of unique evolutionary history and with living genera that have origins deep in the Mesozoic.

## Data Availability

All data are deposited in Dryad [115]. Supplementary material is available online [116].
